# MR thermometry with high precision and temporal resolution by quadratic phase MR fingerprinting

**DOI:** 10.1002/mrm.30546

**Published:** 2025-04-28

**Authors:** Sarah J. Garrow, Kristen Zarcone, Kathryn E. Keenan, Rasim Boyacioğlu, Mark Griswold, William A. Grissom

**Affiliations:** ^1^ Biomedical Engineering Case Western Reserve University Cleveland Ohio; ^2^ National Institute of Standards and Technology Boulder Colorado; ^3^ Department of Radiology Case Western Reserve University Cleveland Ohio

**Keywords:** focused ultrasound, interventional MRI, magnetic resonance fingerprinting, proton resonance frequency shift, temperature imaging, thermometry

## Abstract

**Purpose:**

To map temperature via the proton resonance frequency‐ (PRF‐) shift with a high frame rate and high precision using quadratic RF excitation phase‐magnetic resonance fingerprinting (qRF‐MRF).

**Methods:**

A continuous balanced qRF‐MRF sequence was implemented using a constant low‐flip‐angle excitation with quadratic RF excitation phase increments, which impart sensitivity to resonance frequency changes from heating by repeatedly sweeping the sequence's resonance frequency between −1/(2TR) and +1/(2TR) Hz, while minimizing sensitivity to $T_{1}$ and $T_{2}$. Temperature maps were reconstructed from sliding windows using the conjugate gradient (CG) algorithm, dictionary matching, and conventional PRF temperature calculations using MRF‐synthesized gradient‐recalled echo (GRE) images. Monte Carlo simulations were performed to optimize the sequence. qRF‐MRF temperature precision was compared to acquisition time‐matched 2DFT GRE temperature maps at 3 Tesla in simulations, phantom imaging, and in vivo imaging. The ability to image dynamic temperature changes was validated in a phantom‐focused ultrasound (FUS) heating experiment.

**Results:**

Compared to 2DFT GRE, the optimized qRF‐MRF sequence achieved an 85% reduction in temperature standard deviation in phantom simulation (0.092 vs. 0.014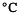), 71% reduction in phantom imaging (0.065 vs. 0.019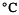), and 55% reduction in vivo (0.321 vs. 0.147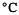). CG MRF reconstruction improved dictionary match inner products ∼ 2× and reduced temperature standard deviation 30% compared to gridding. In FUS heating, qRF‐MRF‐reconstructed heating pattern and temperature curve closely matched the 2DFT results. qRF‐MRF also enabled the reconstruction of linewidth maps.

**Conclusion:**

Continuous low‐flip‐angle qRF‐MRF is capable of temperature imaging using the PRF shift with similar frame rates but higher precision than conventional GRE thermometry.

## 1 INTRODUCTION

Proton resonance frequency shift (PRFS) thermometry is the current standard for MR‐based temperature monitoring in interventional procedures such as laser interstitial thermal therapy (LITT) and MR‐guided focused ultrasound (MRgFUS).^1^ These procedures are FDA‐approved for the treatment of conditions including essential tremor and medically refractory epilepsy. PRFS thermometry is based on the stretching of hydrogen bonds as water heats, which increases the electron shielding of protons from the external magnetic field, yielding a decrease in Larmor frequency with increasing temperature. First described in 1995, real‐time PRFS thermometry for guiding thermal interventions universally uses gradient‐recalled echo (GRE) imaging to measure the frequency shift as a shift in image phase from a baseline preheating state.^2^, ^3^ PRFS thermometry is favored over alternative temperature contrasts such as changes in tissue relaxation times due to the high temporal resolution with which it can be measured, its linearity with temperature, and its relative tissue‐type independence for aqueous tissues.^1^

Since its introduction, many technological advances have been made in PRFS thermometry, which have primarily focused on increasing frame rate and volume coverage and minimizing temperature errors due to motion and respiration.^4^ These advances have been achieved by new image reconstruction and temperature estimation methods, combined with spoiled GRE pulse sequences based on fast imaging scans such as echo‐planar and spiral imaging.^5^, ^6^, ^7^, ^8^ However, few fundamentally new pulse sequence approaches to measure temperature via the PRF shift have been reported. Conventional gradient echo PRFS sequences require relatively long echo times for phase contrast to accrue, with the ideal TE = $T_{2}$*.^3^ In practice, TEs between 10 and 20 ms are common, depending on field strength. However, while sensitivity to temperature increases with increasing echo time, as tissue heats there is a corresponding decrease in SNR due to decreasing $T_{2}$* and increasing $T_{1}$, and long TEs result in long TRs, limiting temporal resolution. The use of low readout bandwidths in long‐echo time GRE sequences to mitigate SNR losses incurred in the use of body coils for signal reception during MR‐guided transcranial ultrasound procedures can further contribute to spatial distortion of temperature maps.^9^

Alternative methods to GRE‐based PRF thermometry have been proposed, such as balanced steady state free precession (bSSFP)‐based PRF thermometry. bSSFP acquisitions have the potential to provide high SNR and increased sensitivity to temperature within more limited temperature ranges.^10^ Mapping the PRF shift with heating from bSSFP images is more complicated than GRE images, as the off‐resonance frequency in bSSFP images has a nonlinear dependence on measured phase changes. One approach to overcome this is to cycle the RF excitation phase to create a signal magnitude‐versus‐RF frequency offset curve, whose peak shifts linearly with temperature changes.^10^ However, this requires the collection of many images at each temperature time point to create the curve, which compromises temporal resolution compared to GRE imaging. Other implementations overcome this by estimating and updating the center frequency at each image in order to use a narrow ($∼10^{\circ }C$) range in which the phase of the bSSFP sequence is a linear and sensitive function of temperature change. This technique is very fast but requires prior estimation or calibration of the range of temperature to be measured, and risks nonlinear phase response and reduced sensitivity outside this range. Similar to multiecho GRE thermometry, multiecho bSSFP thermometry methods calculate temperature from the phase difference between multiple echo times, and in comparison to GRE, principally they provide an SNR increase but are sensitive to banding.^11^, ^12^ Thermometry methods based on MR spectroscopy measure temperature from the separation of peaks from different tissue components. These methods yield absolute thermometry measurements,^13^ but to date, their limited spatiotemporal resolution has constrained their application to static temperature measurement, or to the measurement of very slow thermal changes on the order of minutes.

We report an approach to measuring temperature changes with the PRF shift using quadratic RF excitation phase MR fingerprinting (qRF‐MRF)^14^ with a low flip angle and continuously sweeping RF excitation phase. Instead of relying on a long TE for phase accrual in GRE, qRF‐MRF can quantify off‐resonance changes with heating using a minimum TE with high SNR efficiency and generates high‐precision temperature maps. Previous implementations of MRF thermometry have aimed to measure temperature changes from multiple tissue parameters and have demonstrated good agreement with reference GRE‐based PRFS thermometry measurements.^15^, ^16^, ^17^ However, these acquisitions are based on repeated conventional MRF measurements lasting at least several seconds each with delays in between for longitudinal relaxation, and are therefore not well‐suited to real‐time temperature monitoring. The acquisition proposed in this work instead is designed to continuously measure the PRF shift with temperature at a frame rate similar to GRE thermometry, with minimal sensitivity to changes in other tissue parameters such as $T_{1}$ and $T_{2}$. In the following sections, the sequence and its operating principle are described along with a sliding window temperature map reconstruction. A series of phantom and in vivo experiments are then reported, which assess its intrinsic precision compared to GRE thermometry with matched frame rate and demonstrate its ability to detect temperature changes with focused ultrasound heating.

## 2 THEORY

### 2.1 Pulse sequence

Quadratic RF excitation phase MRF (qRF‐MRF) was first proposed by Wang et al.^14^ for measuring $T_{2}^{*}$ simultaneously with static off‐resonance frequency, $T_{1}$, and $T_{2}$. It works by continuously incrementing the RF excitation phase of a balanced sequence to sensitize signals produced by isochromats to off‐resonance. A quadratic RF excitation phase increment leads to a repeating linear resonance frequency sweep across TRs, and by repeating positive and negative quadratic phase increment periods, Wang et al. achieved a repeating zig‐zag resonance frequency pattern in time that yielded high sensitivity to off‐resonance and intravoxel frequency dispersion.

**FIGURE 1:**
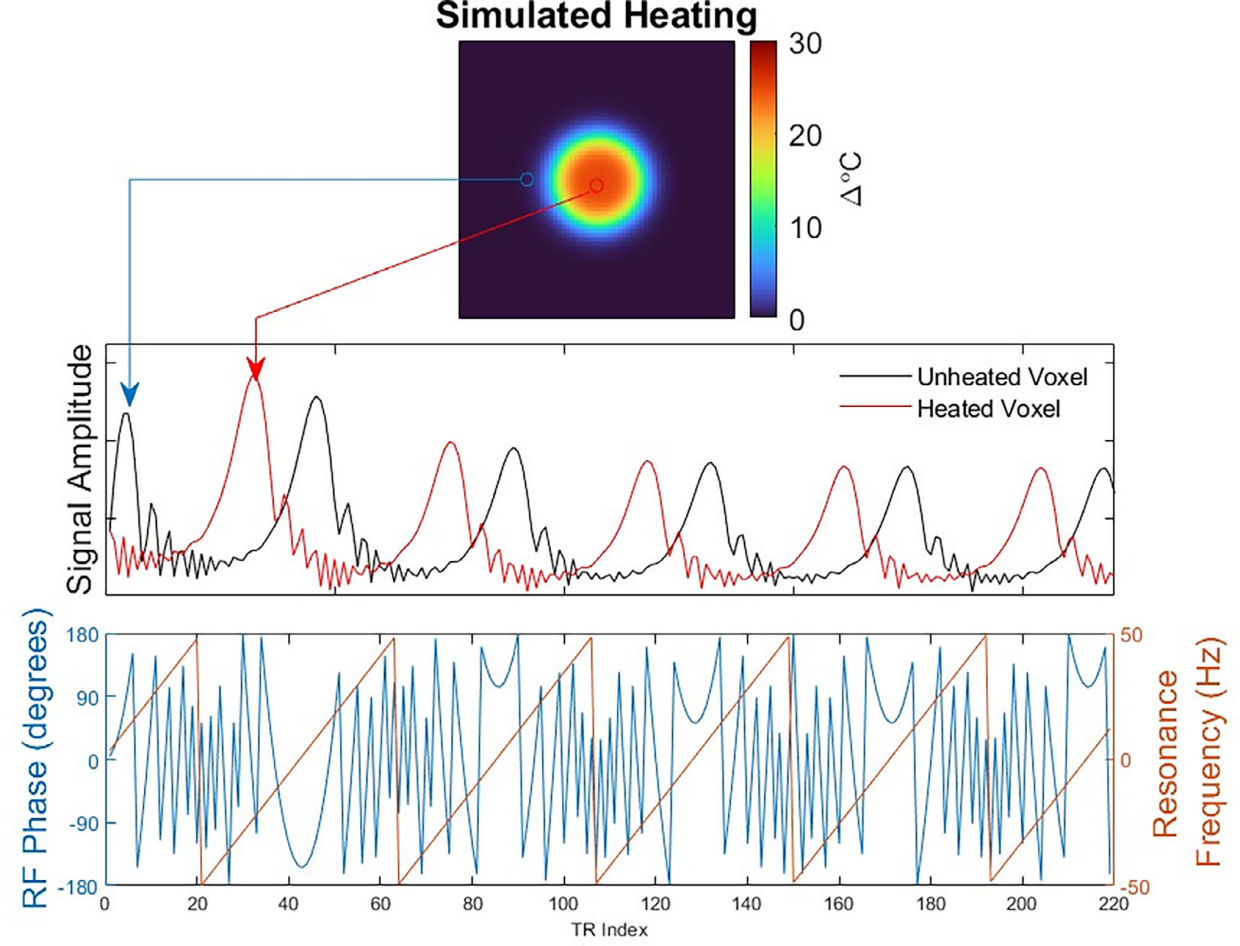
Mechanism of the qRF‐MRF thermometry pulse sequence. Top: A simulated Gaussian hot spot with a peak temperature rise of 25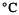 corresponding to a frequency shift of −32 Hz at 3T, in a digital phantom with uniform $M_{0}$, $T_{1}$, and $T_{2}$. Middle: Amplitudes of the simulated signals generated by the phantom at an unheated location and the heated location for the first 220 TR indices of the qRF‐MRF pulse sequence. Bottom: The qRF‐MRF sequence's quadratic RF excitation phase (blue) and linear resonance frequency sweeps (red) for the same TR indices. Each time the sequence sweeps past a resonance frequency, isochromats at that frequency generate a signal peak. Signals at different frequencies peak at different times, enabling differentiation by dictionary matching.

Here, we describe a qRF‐MRF sequence designed to measure only off‐resonance frequency, but continuously and in real time. Figure 1 illustrates the sequence's function by showing signals generated by two voxels in a simulated 2D imaging slice with uniform $M_{0}$, $T_{1}$, and $T_{2}$, but with varying resonance frequencies due to the presence of a Gaussian hot spot. Specifically, one voxel is in an unheated region of the image and has a frequency offset of 0 Hz, while the other voxel lies in the middle of the hot spot and was heated to 25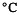 above baseline, corresponding to a PRF shift of −32 Hz at 3T. The proposed qRF‐MRF sequence uses a constant TR of 10 ms and an RF excitation phase of $\phi [n]=4.2n^{2}$ degrees, where n is the TR index, and c=4.2 degrees per TR index‐squared is the RF quadratic phase coefficient. It also uses a low flip angle of 10 degrees to intentionally desensitize the signals to $T_{1}$ and $T_{2}$ and to changes in those parameters with heating. These choices of flip angle and excitation phase are studied further below. The discretized sampling of the quadratic RF excitation phase at a sampling period equal to the sequence's TR corresponds to a repeating resonance frequency sweep between −1/(2TR)=−50 Hz and +1/(2TR)=+50 Hz. We can solve for the resonance frequency at each TR from centered finite differences of ϕ[n], as

(1) $$\begin{array}{ll} f\left[n\right] & =\frac{1}{360}\frac{c{\left(n+1\right)}^{2}-c{\left(n-1\right)}^{2}}{2\text{TR}} \end{array}$$

(2) $$\begin{array}{ll} & =\frac{1}{360}\frac{cn^{2}+2cn+c-cn^{2}+2cn-c}{2\text{TR}} \end{array}$$

(3) $$\begin{array}{ll} & =\frac{1}{360}\frac{2cn}{\text{TR}} \end{array}$$

(4) $$\begin{array}{ll} & =\frac{cn}{180\text{TR}}, \end{array}$$

where the factor 1/360 converts units of degrees to phase cycles. To determine the period N (units of TR indices) of the frequency sweep, we note that the frequency sweep will repeat every 1/TR Hz, so its period can be determined by solving

(5) $$\frac{cN}{180\text{TR}}=\frac{1}{\text{TR}},$$

yielding:

(6) $$N=\frac{180}{c}.$$

**FIGURE 2:**
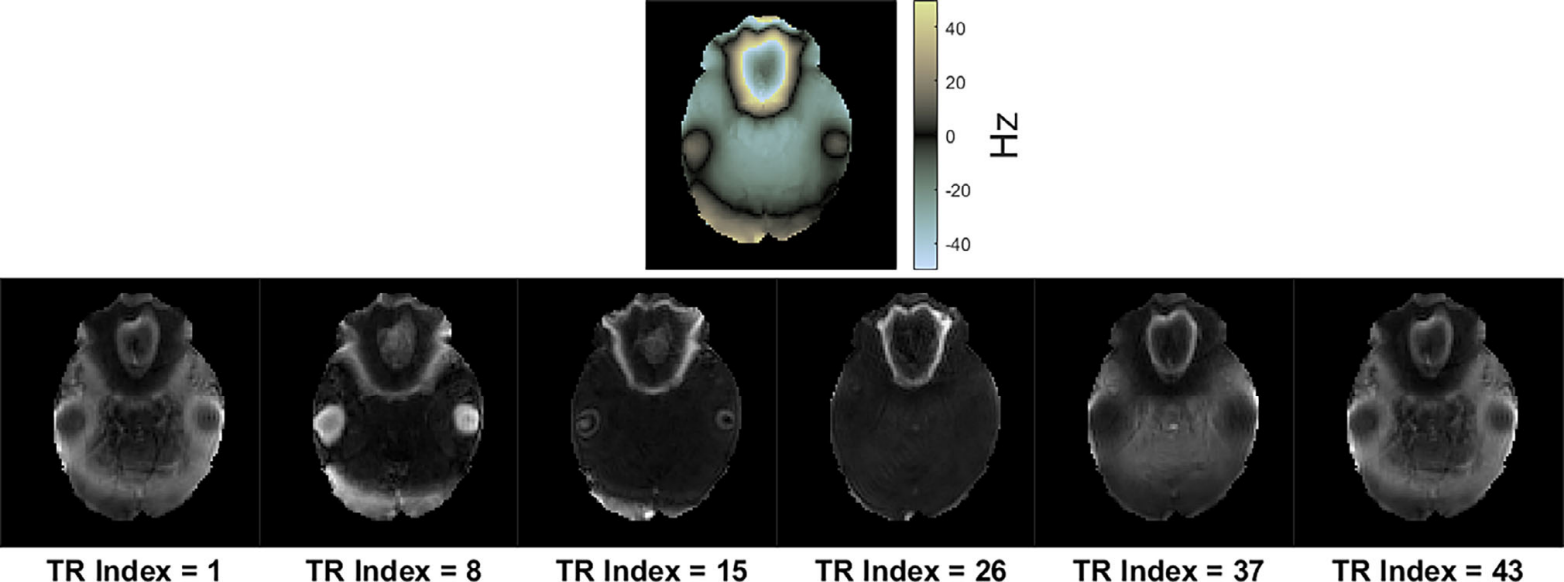
(A) A frequency map reconstructed from one time window in Volunteer 1's inferior slice. (B) Images at different TRs for the frequency map shown in (A). The signal evolves with the contours of the off‐resonance map as the sequence's resonance frequency sweeps between −50 and +50 Hz.

Applying Equation 6, the frequency sweep period of the qRF‐MRF sequence shown in Figure 1 with 1/TR=100 Hz and c=4.2 degrees per TR index‐squared has a period of N=180/4.2≈42.9 TRs, which is reflected in the distance between consecutive signal peaks for each voxel. This is the pulse sequence used throughout this work. Figure 2B illustrates how signal across a brain slice peaks at different times over 43 TRs, which tracks the contours of the slice's off‐resonance map shown in Figure 2A.

### 2.2 Temperature map reconstruction: Frequency mapping

To reconstruct dynamic temperature change maps from qRF‐MRF data, a Bloch‐simulated dictionary of the entire pulse sequence is generated which principally spans off‐resonance frequencies from −1/(2TR) Hz to +1/(2TR) Hz, but may also span linewidths, $T_{1}$, $T_{2}$, and any other desired parameters. The measured data are then divided into few‐second‐long windows that are short enough that temperature may be approximated as constant, and the corresponding few‐second‐long windows are extracted from the dictionary. Each window is processed independently of the others. Within a window, parameter map reconstruction follows a conventional MRF reconstruction procedure,^18^ in which images are reconstructed for every TR and each voxel's signal is matched to the corresponding window from the dictionary to generate a frequency map.

**FIGURE 3:**
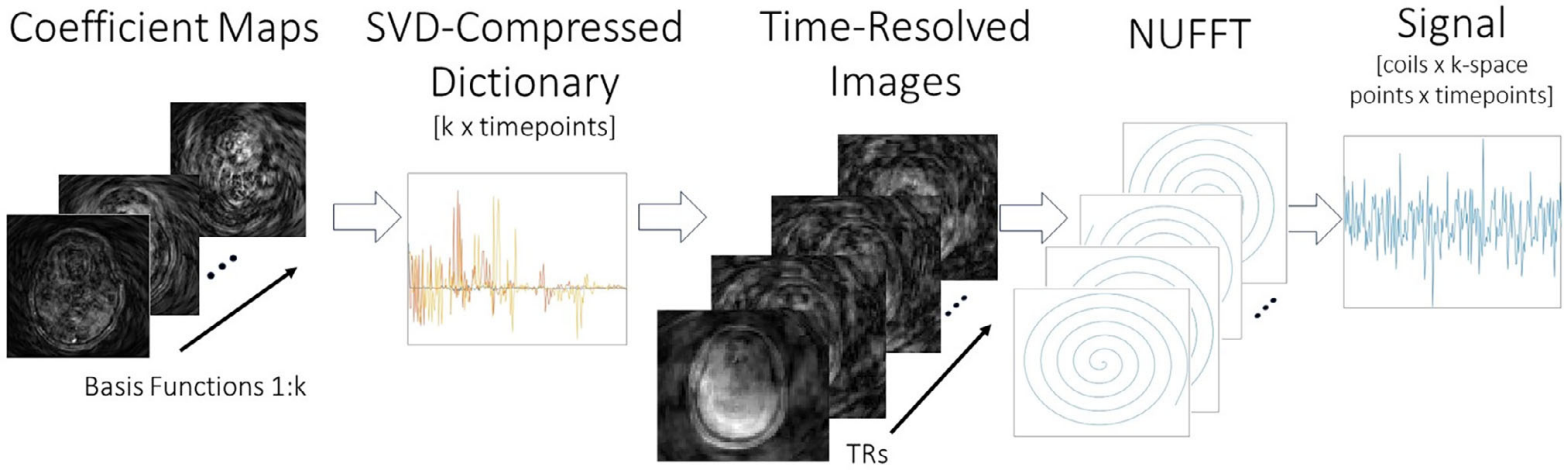
Forward model for conjugate gradient image reconstruction of each window's images. The model inputs coefficient maps, one map for each temporal basis function in the window's SVD‐compressed dictionary. The product of the coefficient maps and the dictionary yields time‐resolved images across all TR indices in the window. Each of these images is multiplied by the coil sensitivity maps and is nonuniform fast Fourier transformed to obtain predicted k‐space signal for each TR index.

An iterative reconstruction procedure was further implemented, which was based on the linear forward model illustrated in Figure 3. For each time window, an SVD‐compressed dictionary was calculated^19^ with k elements. This compressed dictionary was used to solve for coefficient maps from the data of that time window. At each iteration of the reconstruction, the current estimates of the coefficient maps were multiplied back into the SVD‐compressed dictionary, which expanded the compressed maps to time‐resolved images, one for each TR in the window. To relate those time‐resolved images back to the multicoil k‐space signals for the next iteration, they were duplicated across coils, multiplied by the coil sensitivity maps, and the nonuniform FFTs (NUFFTs) of the coil images were calculated. This model was implemented as a linear operator and passed to a conjugate gradient (CG) algorithm that solved for the coefficient maps, with no additional regularization. The time‐resolved images from the last iteration are used for temperature map reconstruction.

### 2.3 Temperature map reconstruction: Temperature calculation

**FIGURE 4:**
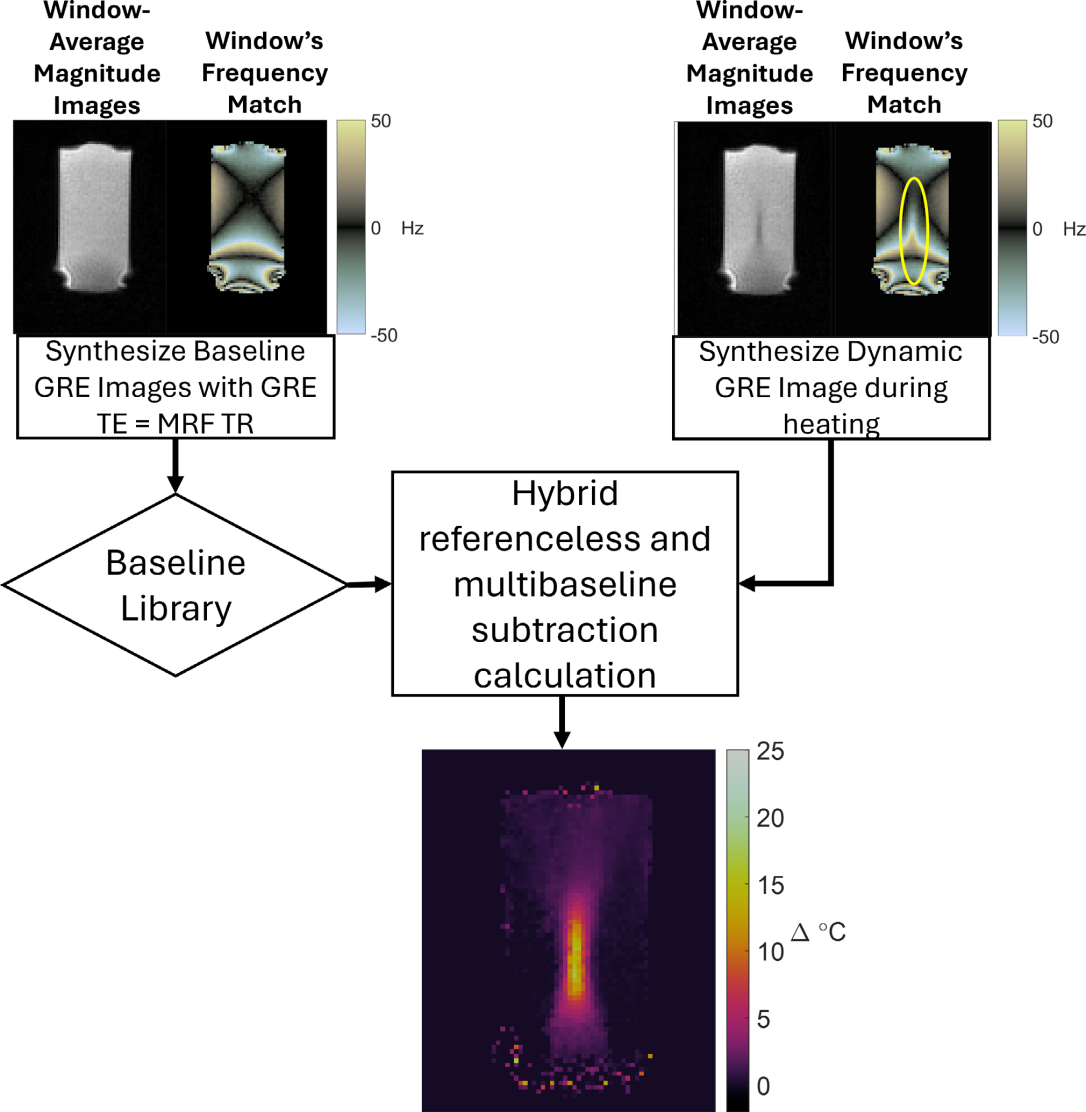
GRE image synthesis and temperature map reconstruction using the hybrid multibaseline and referenceless subtraction algorithm. The images and maps are from the focused ultrasound heating experiment (Figure 9). For each window, the matched frequency maps and the magnitude of the window's average image are combined to form a synthetic GRE image using Equation (7). A set of synthesized baseline GRE images forms a baseline library to remove bulk motion, and a dynamic GRE image is synthesized for the heating window. These are then input to the algorithm, which yields a sparse temperature phase shift map that is converted to temperature change using Equation (8).

To leverage an existing motion‐ and respiration‐robust temperature mapping method such as the hybrid multibaseline and referenceless thermometry algorithm,^20^ temperature was calculated from the reconstructed time‐resolved images and frequency maps following the procedure illustrated in Figure 4. The procedure used synthesized GRE images, which were calculated according to

(7) $$I_{GRE}\left(\vec{x}\right)=\left|I_{\text{mean}}\left(\vec{x}\right)\right|e^{ı2\pi TR\Delta f\left(\vec{x}\right)},$$

where $I_{\text{mean}}\left(\vec{x}\right)$ is the mean image across the time points in a window, and $\Delta f\left(\vec{x}\right)$ is the window's frequency map. It is required to use the MRF sequence's TR rather than its TE for the synthesized image since its TR determines the frequencies at which aliasing occurs: in qRF‐MRF, wrapping occurs at −1/(2TR) and +1/(2TR) Hz, rather than at −1/(2TE) and +1/(2TE) Hz as in a conventional GRE sequence.

GRE images were synthesized for several windows prior to heating and collected into a baseline library, which are one input to the hybrid algorithm. The other input to the algorithm is a synthesized dynamic GRE image for a window during heating. The hybrid algorithm then reconstructs a temperature map for the dynamic heating window by fitting an image model to that window's synthesized image. The image model comprises a weighted average of the synthesized baseline images, which is phase‐shifted by a low‐order polynomial phase shift map that models respiratory and other sources of bulk phase shifts, and a spatially sparse phase shift map $\Delta \theta \left(\vec{x}\right)$ representing temperature changes. Further algorithmic details are provided in Reference 20. The reconstructed temperature phase shift map produced by the hybrid algorithm is converted to a change in temperature using

(8) $$\Delta T\left(\vec{x}\right)=-\frac{\Delta \theta \left(\vec{x}\right)}{\gamma \alpha \text{TR}B_{0}},$$

where γ is the gyromagnetic ratio and α=−0.01 ppm/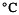 is the PRF change coefficient for aqueous tissue. This procedure required no modifications to the hybrid algorithm.

## 3 METHODS

### 3.1 Pulse sequence implementation

The qRF‐MRF thermometry sequence was implemented on a 3T Vida scanner (Siemens Healthineers, Erlangen, Germany) using a 64‐channel receive coil for all experiments except the phantom heating experiment, which used a 20‐channel receive coil to accommodate the phantom and ultrasound transducer. In each scan, coil elements distant from the imaged slice were switched off. The sequence used a constant TR of 10 ms and TE = 1.8 ms, a constant flip angle = 10°, and a quadratically increasing RF excitation phase of $4.2n^{2}$ degrees, where n is the TR index. A 6 ms‐long spiral readout with twelve interleaves and receiver dwell time 2 μs was used for spatial encoding. The spirals were uniformly sampled in the radial dimension and were acquired in linear order. For reconstruction, the spirals were measured using a modified Duyn's method.^21^ In all experiments, images were also acquired using a conventional Cartesian 2DFT GRE thermometry sequence with TE = 12 ms, TR = 17 ms, and 40 kHz bandwidth. The flip angle was set to Ernst angle values for each medium: 13° for the functional Biomarker Imaging Research Network (fBIRN) phantom experiments,^22^ 19° for agar‐graphite phantom heating experiments, and 30° for in vivo experiments. A Cartesian 2DFT sequence was chosen as the benchmark for comparison. Both the qRF‐MRF and 2DFT sequences were written in Pulseq,^23^ with the same excitation pulse shape so that their slice profiles matched. Both sequences imaged a 256 × 256 mm^2^ FOV with ×2× mm^3^spatial resolution. Frequency, temperature, and linewidth maps were visualized using MRF colormaps,^24^ and standard deviation maps were visualized using Crameri colormaps.^25^

### 3.2 Temperature map reconstruction

For reconstruction from simulated and experimental data, a dictionary was calculated using a Bloch equation simulation of the pulse sequence with 4096 frequency points ranging from −50 to +50 Hz, short and long $T_{1}$/$T_{2}$ pairs ($T_{1}$ = [250 1250] ms, $T_{2}$ = [10 50] ms), and 17 Gaussian linewidths from 1 to 49 Hz, unless specified otherwise. Unless stated otherwise, temperature maps were reconstructed using the CG method (MATLAB's lsqr() (Mathworks, Natick, MA, USA) using min‐max NUFFT's^26^) with 10 iterations, no regularization, and k=15 temporal basis functions from 2.2 s‐long windows of nonoverlapping data (220 TRs), to match the 2DFT scan time.

### 3.3 Simulations: Sequence optimization and temperature precision versus 2DFT

Monte‐Carlo simulations were performed to optimize the qRF‐MRF sequence and compare its precision to the 2DFT scan described above. A uniform circular object with a 64 voxel diameter was defined on a 128 × 128 grid. The object's relaxation times were set between grey and white matter at 3T: $T_{1}$ = 825 ms, $T_{2}$ = 70 ms, and $T_{2}$* = 46 ms. Its frequency map was set to a uniform 0 Hz. To simulate a qRF‐MRF scan of the object, a qRF‐MRF signal was generated from the 0 Hz frequency offset entry of the dictionary for 660 TRs, the second 220 (2.2 s) of which corresponds to the baseline frequency map, and the third 220 of which corresponded to the heated frequency map. The first 220 TRs were discarded to remove transient effects. The signals were replicated to all spatial locations in the object to obtain an image for each TR. k‐space data for each TR were calculated using an NUFFT applied to each TR's image, and independent zero‐mean complex‐valued Gaussian noise was added to the k‐space data after scaling by a factor that produced a signal‐to‐noise ratio of 112 in the 2DFT images (described below), and also by the square root of the ratio of the 2DFT and qRF‐MRF dwell times, to account for the difference in readout bandwidth between the 2DFT and qRF‐MRF scans. The data generation was repeated for 50 noise realizations. Images were reconstructed from the noisy data using the CG method or gridding with density compensation. To minimize the effect of dictionary frequency resolution on measured temperature precision, the dictionary for final matching was finely sampled with 8192 points over a ±50 Hz range, corresponding to a temperature resolution of 0.0095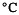. After dictionary matching, a temperature map was calculated for each realization by single baseline subtraction and a standard deviation map was calculated across realizations.

To optimize the qRF‐MRF sequence's flip angle and quadratic RF excitation phase curvature (c), the above Monte‐Carlo simulation was repeated for flip angles of 5, 10, 15, and 20 and *c* = 1.05, 2.1, 4.2, and 8.4 degrees per TR index‐squared (16 total parameter combinations). The TE and TR were set the same as reported above.

To compare qRF‐MRF temperature precision to the 2DFT scan, baseline and heating 2DFT images were calculated after scaling the object to a steady‐state spoiled GRE signal amplitude^27^:

(9) $$M_{xy}^{\text{GRE}}=\frac{\sin\left(\alpha \right)(1-e^{-\text{TR}/T_{1}})}{1-\cos\left(\alpha \right)e^{-\text{TR}/T_{1}}}e^{-\text{TE}/T_{2}^{*}},$$

where the flip angle α was set to the Ernst angle, $\alpha =11.6^{\circ }$. Fifty independent complex‐valued zero‐mean Gaussian noise realizations were added to the baseline and heating images for a signal‐to‐noise ratio of 112, which was the value measured from the phantom precision imaging experiment described below. A temperature map was calculated for each noise realization using baseline subtraction, and a standard deviation map was calculated across realizations.

### 3.4 Within‐window temperature change simulation

The qRF‐MRF sequence samples the middle of k‐space at each TR/timepoint and fully covers k‐space multiple times within each time window, leading to the expectation that it will reconstruct the average temperature change within a time window. To test this, a signal was Bloch‐simulated for a constant 5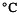 temperature rise, and its inner product was calculated with each entry of a dictionary spanning the frequency/temperature range for the same time window, where the inner product of a dictionary entry and the signal is a time point‐by‐time point multiplication and sum of the signal with the dictionary entry, and measures how much the signal correlates with the dictionary entry. This was compared with a signal that was Bloch‐simulated for a rapid linear 0–10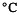 temperature rise over the time window, whose dictionary inner products were also recorded.

### 3.5 Phantom temperature precision experiment

The Monte Carlo temperature simulation results were verified in a phantom experiment. An unheated fBIRN phantom whose $T_{1}$ and $T_{2}$ reflect human brain tissue was scanned for two minutes using 2DFT and qRF‐MRF sequences. A single linewidth of 1 Hz was used for reconstruction in the uniform phantom. For qRF‐MRF data used in this experiment, the dictionary was constructed with a finely sampled frequency resolution of 8192 points over a ±50 Hz range to match that used in the phantom precision simulation. To compensate scanner field drift, temperature maps were reconstructed using the hybrid multibaseline and referenceless algorithm for both 2DFT and qRF‐MRF temperature maps, using eight baseline images and a first‐order polynomial to remove errors due to $B_{0}$ drift and time‐varying gradient eddy currents. Through‐time temperature standard deviation maps were calculated from the reconstructed temperature maps.

### 3.6 In vivo temperature precision experiment

In order to evaluate the robustness and precision of the sequence in vivo, two healthy adult volunteers, one male and one female, were scanned without heating. Prior to imaging, written informed consent was obtained from each participant in accordance with the Institutional Review Board at University Hospitals. Two axial slices were imaged in each volunteer at two different locations, one higher slice that cut through the hippocampus, which is a common target for laser thermal therapies to treat epilepsy,^28^ and one lower slice that cut through the thalamus, which is the target for focused ultrasound thalamotomies to treat movement disorders.^29^ At each location, each volunteer was scanned twice using both the qRF‐MRF sequence and the 2DFT sequence. The total scan durations were matched between 2DFT and qRF‐MRF, with each one lasting 2 minutes, corresponding to 54 time points. 2DFT and qRF‐MRF temperature maps were reconstructed using the hybrid multibaseline and referenceless algorithm with 8 baseline images and a second‐order polynomial to remove errors caused by $B_{0}$ drift and time‐varying gradient eddy currents, as well as those caused by physiological variations such as respiration. Through‐time temperature standard deviation maps were calculated for each method and slice. To evaluate temperature precision as a function of window width, qRF‐MRF temperature map reconstructions were repeated with 1.1 and 3.3 s‐long windows, in addition to the 2DFT‐matched 2.2 s width.

### 3.7 Phantom heating experiment

To confirm that qRF‐MRF can provide dynamic temperature mapping, focused ultrasound heating was performed in a 1% w/v agar/4% w/v graphite phantom. An 850 kHz focused ultrasound transducer (model H115‐MR, Sonic Concepts, Bethel, WA, USA) was used to heat the phantom for 45 s. The transducer was driven by a 150 W/55 dB gain amplifier (model A150, Electronics and Innovation, Rochester, NY, USA), with a 250 mV peak‐to‐peak 850 kHz sinusoidal signal on its input. Heating was repeated while imaging with both the 2DFT and qRF‐MRF sequences, with fifteen minutes in between to allow the phantom to cool. The phantom was oriented so that the ultrasound propagation direction coincided with the scanner's y‐direction, and the 2DFT and qRF‐MRF sequences scanned the same transverse slice containing the focus. $T_{1}$ and $T_{2}$ values of the phantom were measured to be 300 ms and 45 ms, respectively, using an MRF scan.^30^ These values were then used in the dictionary for reconstruction. 2DFT and qRF‐MRF temperature maps were reconstructed using the hybrid multibaseline and referenceless algorithm with eight baseline images and a first‐order polynomial to remove errors due to $B_{0}$ drift and time‐varying gradient eddy currents.

## 4 RESULTS

### 4.1 Temperature precision simulations

Table 1 reports the qRF‐MRF temperature standard deviations measured from the Monte‐Carlo simulations for each quadratic RF excitation phase curvature and flip angle, for a 2.2‐s window width/220 TRs. The standard deviation is lowest at a flip angle of $10^{\circ }$ and a curvature of c=4.2 degrees per TR index‐squared, though the curvature has a bigger effect than the flip angle, except for the smallest curvature. These results motivate the use of this flip angle and curvature in the rest of the work.

**FIGURE 5:**
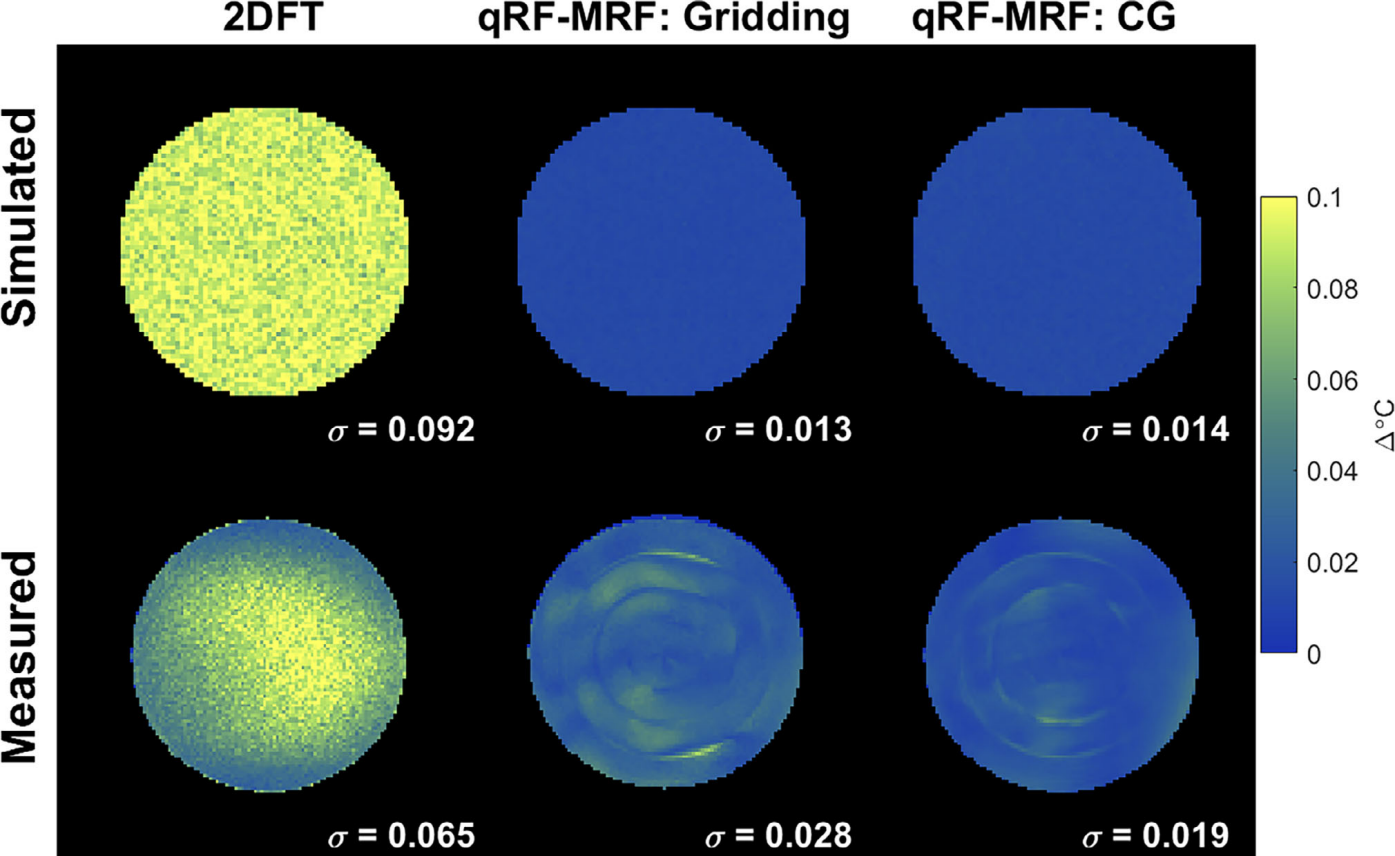
Top row: Monte‐Carlo‐simulated temperature standard deviation maps for the 2DFT and qRF‐MRF scans, where qRF‐MRF data were reconstructed using gridding or CG. The qRF‐MRF window width was 2.2 s to match the 2DFT scan time. The overall standard deviations are reported in white, in units of 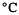. Bottom row: Experimental 2DFT and qRF‐MRF temperature standard deviation maps over two minutes in an unheated fBIRN phantom. The qRF‐MRF window width was 2.2 s to match the 2DFT scan time. The overall standard deviations are reported in white, in units of degrees Celsius.

**TABLE 1:** qRF‐MRF temperature standard deviations (in degrees Celsius) calculated from Monte–Carlo simulations of a circular object (Figure 5) using gridding reconstruction, across ranges of flip angles and quadratic phase curvatures.

	1.05n 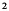	2.1n 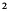	4.2n 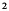	8.4n 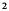
5°	0.0178	0.0159	0.0151	0.0155
10°	0.0179	0.0145	**0.0130**	0.0135
15°	0.0280	0.0178	0.0137	0.0156
20°	0.0478	0.0163	0.0160	0.0190

*Note*: n is the TR index. The combination with the lowest standard deviation (10° flip angle and $c=4.2^{\circ }$ per TR index‐squared) is bolded and was used in the rest of this work.

Figure 5 compares 2DFT versus qRF‐MRF through‐time temperature standard deviation maps using the above RF excitation phase curvature and flip angle for qRF‐MRF. Using a gridding reconstruction, the qRF‐MRF temperature maps had 86% lower temperature standard deviation than 2DFT, where the 2DFT standard deviation was 0.092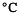, compared with 0.013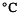 for qRF‐MRF. The CG reconstruction had a similar improvement compared with 2DFT, reducing the error by 85% to 0.014. This number is very low (less than twice the temperature precision of the dictionary) but slightly higher than the gridding result, likely due to the constrained nature of the CG reconstruction. It may be reduced to a lower level by further tuning of the SVD dictionary compression. Supporting Information Figure S6 further shows simulation results using a longer 2DFT TE = $T_{2}^{*}$ and time‐matched qRF‐MRF window widths. With this SNR‐optimal TE^3^ and commensurately longer 7.68‐s scan time, 2DFT's precision improves to 0.027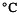. This is still more than twice the error of qRF‐MRF with the same window width and almost twice the error of qRF‐MRF at the much shorter 2.2‐s window width.

### 4.2 Within‐window temperature change simulation

Supporting Information Figure S1 compares dictionary inner products for a constant 5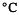 temperature change over a 220‐timepoint window, with inner products for a rapid 0–10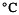 temperature change over the same window. Because qRF‐MRF uses spiral readouts that sample the middle of k‐space each TR and that collect a full k‐space dataset multiple times within each window, the resulting inner products are equally weighted by each time point in the window, so the inner product spectrum becomes wider in the 0–10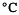 rise case but the peak remains at the same temperature. This is in contrast to a 2DFT sequence in which the middle of k‐space is sampled once in the middle of a time window, so reconstructed temperature is more heavily weighted to that time point.

### 4.3 Phantom temperature precision experiment

Figure 5 also shows experimental temperature standard deviation maps for 2DFT (2.2 s per image) and qRF‐MRF (2.2 s window width/220 TRs, and gridding and CG reconstructions). The center of the 2DFT map has higher standard deviation than the periphery, possibly due to lower receive sensitivity in the middle of the phantom. The qRF‐MRF maps contain ring artifacts which were the likely result of residual k‐space trajectory errors or $B_{0}$ eddy currents not present in the simulations. Supporting Information Figure S2 shows qRF‐MRF frequency maps at regular TR intervals through the scan. Frequencies ranged from −7 to +10 Hz with a mean of 2.3 Hz and a standard deviation of 2.4 Hz, corresponding to only 6% of a phase cycle across the qRF‐MRF 6 ms spiral readout. This indicates that off‐resonance did not likely significantly contribute to temperature error in these scans. The overall gridding and qRF‐MRF standard deviations were 57% and 71% lower than 2DFT, respectively.

### 4.4 In vivo temperature precision

**FIGURE 6:**
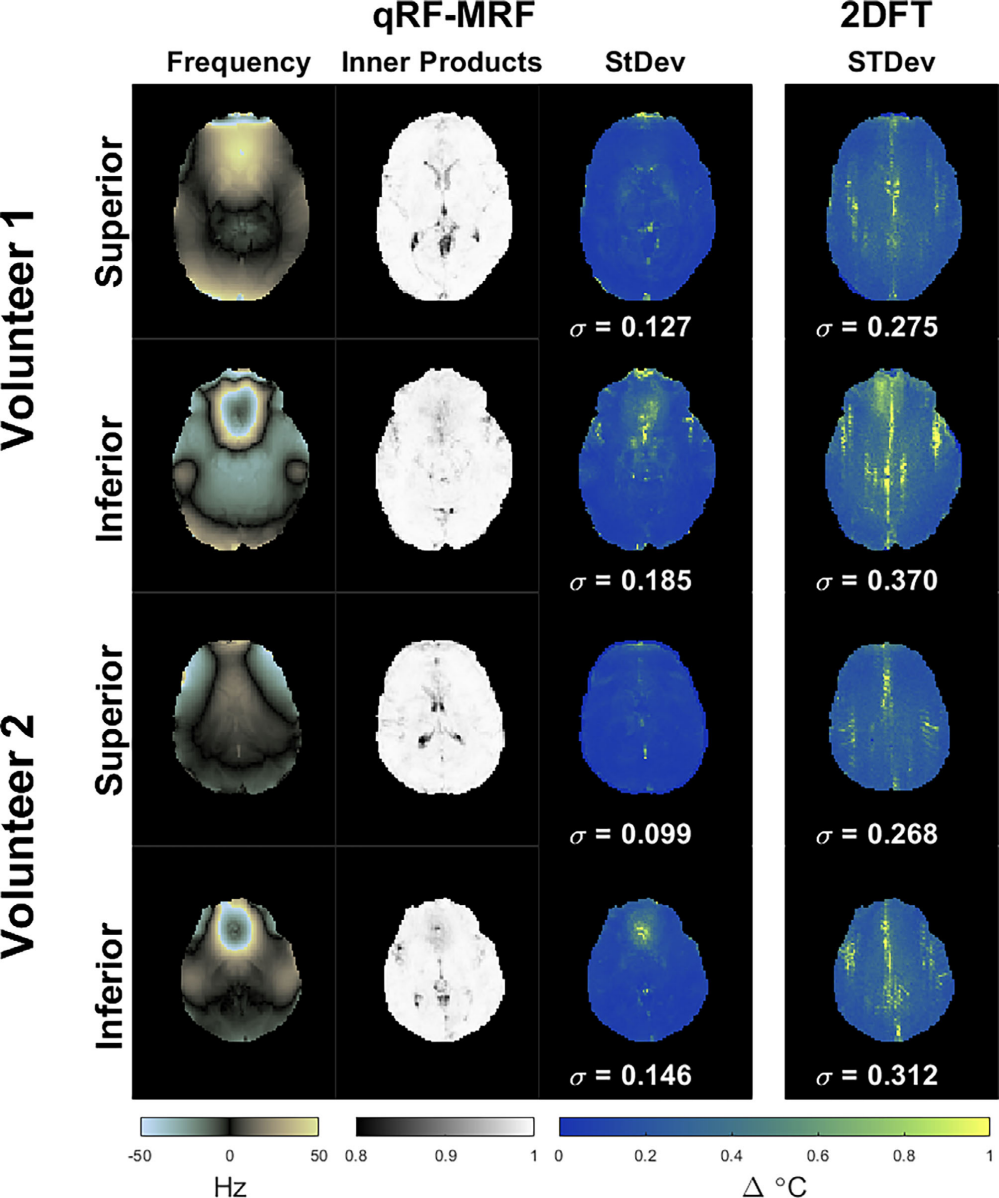
Two‐volunteer, two‐slice temperature mapping without heating, for precision assessment. The qRF‐MRF window width was 2.2 s to match the 2DFT scan time, and the CG method was used for qRF‐MRF reconstruction. A representative single‐window qRF‐MRF frequency map (first column) and inner product map (second column) are shown for each slice. qRF‐MRF through‐time temperature standard deviation maps are shown in the third column, and the fourth column shows 2DFT through‐time temperature standard deviation maps for the same slices. The overall standard deviations are reported in white, in units of degrees Celsius.

Figure 6 shows the in vivo temperature precision results. A representative single‐window off‐resonance frequency map is shown for each qRF‐MRF slice (CG reconstruction), as well as its inner product map. The inner products were overall high, all averaging over 0.98 with a standard deviation under 0.01, despite, including only two $T_{1}$‐$T_{2}$ pairs in the dictionary. The through‐time temperature standard deviation maps for each slice are shown for qRF‐MRF and 2DFT in each slice. Supporting Information Figure S3 compares gridding and CG reconstruction in Volunteer 2's superior slice. Gridding reconstruction produced inner products that were half those of the CG reconstructions on average, and had 30% higher temperature standard deviation. Supporting Information Figure S4 compares the two repeated scans of each slice. The repetitions were close, with six of the eight repetitions having standard deviations within 0.02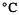 of each other. Overall, averaging across volunteers, slices, and repetitions, the qRF‐MRF standard deviations were 55% lower than the 2DFT standard deviations.

**FIGURE 7:**
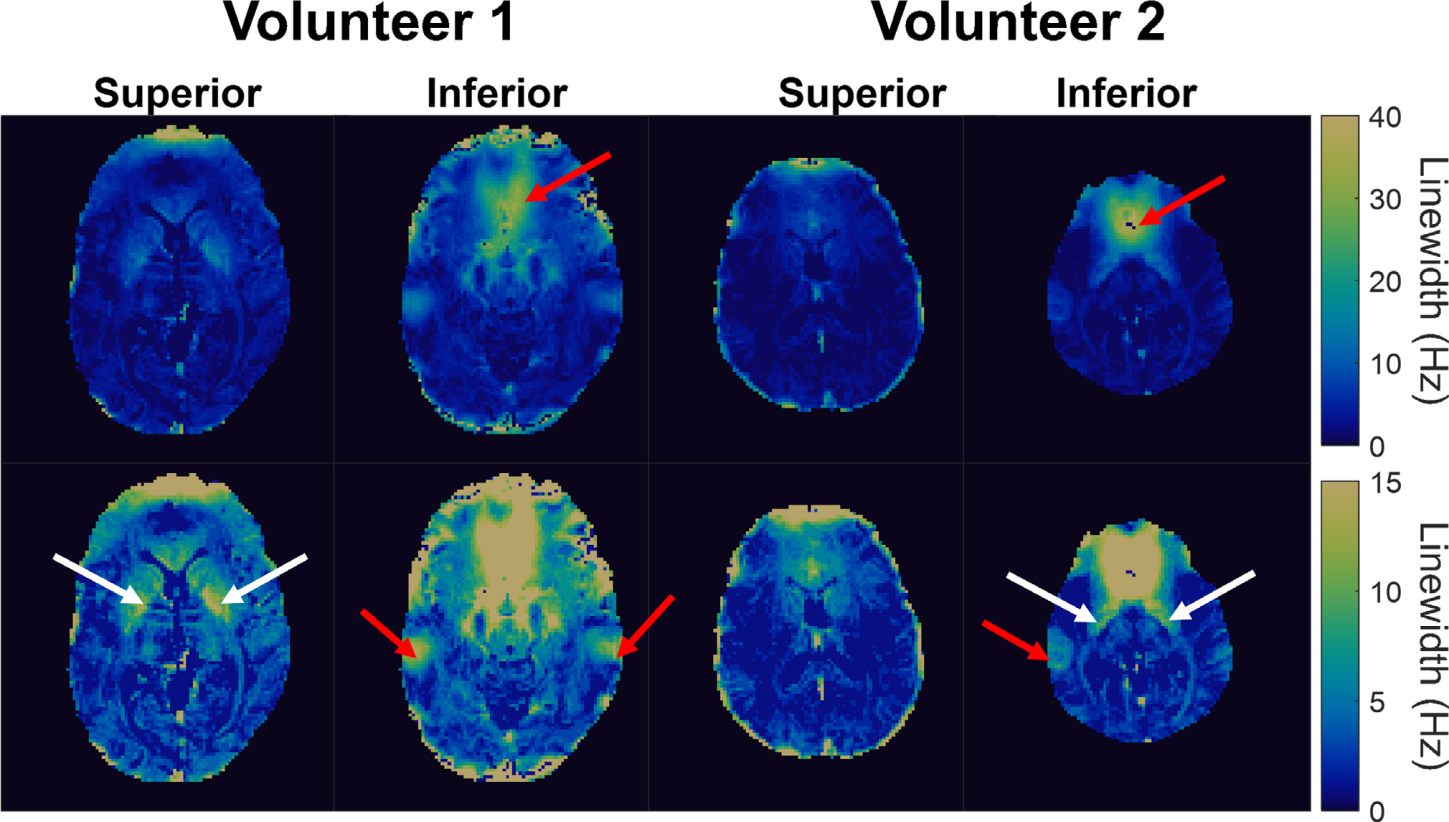
Linewidth maps reconstructed from one time window of the qRF‐MRF scans in each slice. The maps reveal areas of high susceptibility above the sinuses and ear canals (red arrows), as well as structures such as the putamen (white arrows) that are high in iron.

Representative single time‐window linewidth maps are shown for each slice in Figure 7. To obtain more spatially continuous maps for display, these maps were generated using a finer linewidth sampling of 1 Hz steps from 1 to 65 Hz. Regions above the sinuses and ear canals have wide linewidths due to off‐resonance gradients through those voxels (red arrows), and iron‐rich structures such as the putamen also have wide linewidths (white arrows). Note that regions with wide linewidths correspond to regions with the highest temperature standard deviation and the lowest correlation in Figure 6, suggesting that temperature precision may be further improved by improving the linewidth model in the dictionary calculation.

**FIGURE 8:**
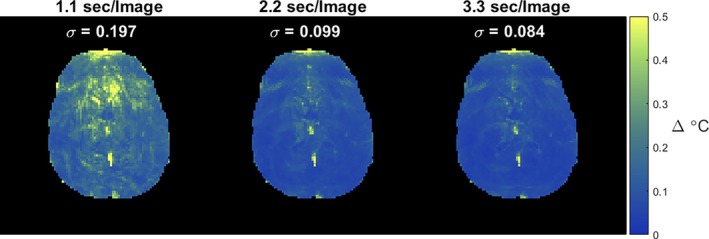
qRF‐MRF through‐time temperature standard deviation maps across window widths for Volunteer 2's superior slice. The overall standard deviations are reported in white, in units of degrees Celsius.

Figure 8 compares through‐time temperature standard deviation maps for different window widths, for Volunteer 2's superior slice. Temperature uncertainty is reduced by half moving from 1.1 to 2.2 s, and is minimally improved moving from 2.2 to 3.3 s. There are likely competing effects that arise for longer window widths: temperature uncertainty is reduced due to increased signal averaging but is likely offset somewhat by physiological variations, in particular as the window contains an increasing proportion of the respiratory period.

### 4.5 Phantom heating experiment

**FIGURE 9:**
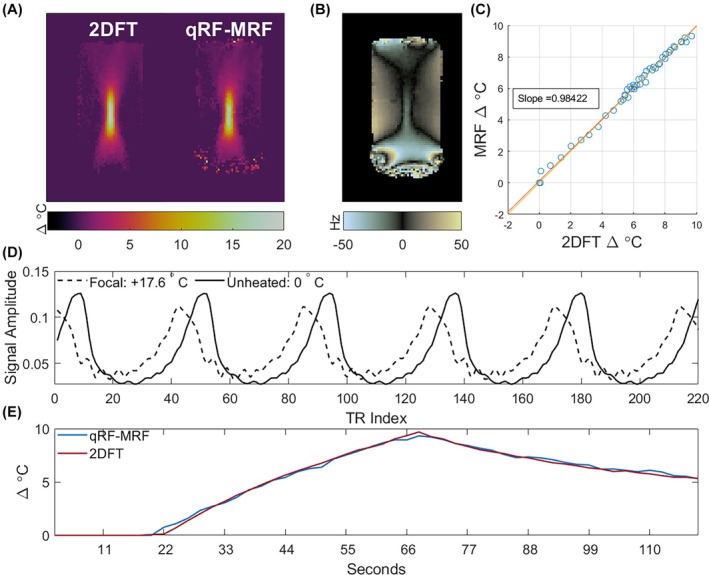
Focused ultrasound (FUS) phantom heating experiment. (A) Temperature maps at peak heat during FUS heating of the phantom using 2DFT and qRF‐MRF scans. The transducer was positioned at the bottom of the FOV, firing upward. (B) The qRF‐MRF frequency map at the same time window as shown in (A). The hot spot is visible on top of background static off‐resonance. (C) Scatter plot of qRF‐MRF versus 2DFT temperatures in a ten‐voxel ROI centered on the focus. A unit‐slope line (yellow) and a line fit to the data (red) are plotted, where the latter had a slope of 0.98. (D) Reconstructed qRF‐MRF signal timecourses of the middle focal and an unheated voxel over TR indices of the window shown in (A). (E) Temperature rises versus time in a ten‐voxel focal ROI.

Figure 9A shows 2DFT and qRF‐MRF temperature reconstructions from the focused ultrasound heating experiments at peak heat, where the qRF‐MRF window width was 2.2 s to match the 2DFT frame rate. The two scans show the same bow‐tie heating pattern. The horizontal full widths‐at‐half maximum of the focus were 7 mm for qRF‐MRF versus 7.4 mm for 2DFT, and the vertical full widths‐at‐half maximum were 36.8 mm for qRF‐MRF versus 35.6 mm for 2DFT. Supporting Information Figure S5 shows magnitude images for 2DFT versus qRF‐MRF from a single time window. The qRF‐MRF SNR measured from those images was 74% higher than the 2DFT SNR. Figure 9B shows the matched qRF‐MRF frequency map at peak heat, from which the qRF‐MRF temperature map in (A) was derived. Figure 9C shows a scatter plot of qRF‐MRF temperature versus 2DFT temperature over the course of the scans in a ten‐voxel ROI centered on the focus. The two match well with a linear fit slope of 0.984, reflecting the same sensitivity to heating. While these temperature reconstructions assumed a PRF change coefficient of −0.01 ppm/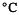, which may not be the most accurate value for this phantom, the methods' matched sensitivity indicates that errors in coefficient would only linearly scale both methods' temperature maps. Figure 9D plots qRF‐MRF signals in the focus (dotted line) and in an unheated voxel (solid line). The peaks of the heated voxel's signal are shifted relative to the unheated voxel due to its −22.5 Hz frequency shift caused by 17.6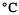 of heating. The heated voxel's signal has slightly lower amplitude, likely due to increased $T_{1}$ and/or decreased $T_{2}$ with heating. The average inner product in a centered 20 × 20‐voxel ROI was 0.90 at peak heating. Figure 9E plots mean temperature versus time in a ten‐voxel ROI covering the focus in the 2DFT and qRF‐MRF maps. The curves follow similar dynamics with a peak difference of 0.37 $\Delta^{\circ }$ C.

## 5 DISCUSSION

This work reported a new approach to measuring temperature changes with the PRF shift using quadratic RF excitation phase MR fingerprinting. By repeatedly sweeping its RF excitation phase and using a low‐flip angle excitation, the proposed balanced sequence provides temperature maps at a frame rate commensurate with that of conventional GRE, but with much improved temperature precision due to its balanced rather than spoiled nature, its lack of a steady state, and its sampling of the middle of k‐space each TR and full sampling of k‐space several times within each window (every 12 TRs in the present implementation). Phantom imaging confirmed this improvement in baseline precision, while in vivo imaging confirmed that a large reduction in temperature errors persists in vivo, despite errors due to respiration and other physiological effects that affect all PRF‐shift thermometry methods. A CG reconstruction was implemented for qRF‐MRF that (compared to gridding) reduced errors due to aliasing artifacts in phantoms and in vivo, and it was shown that qRF‐MRF temperature maps can be reconstructed using existing processing methods developed to mitigate motion, such as the hybrid multibaseline and referenceless algorithm. Finally, phantom‐focused ultrasound heating experiments confirmed that qRF‐MRF can image dynamic temperature changes and provides measurements that agree well with conventional 2DFT GRE‐based PRF‐shift thermometry with matched temperature sensitivity and hot spot dimensions.

MRF thermometry has been previously reported using multiparametric conventional MRF and qRF‐MRF acquisitions at much slower frame rates.^15^, ^16^, ^17^ The qRF‐MRF scan used in this work was intentionally desensitized to $T_{1}$, $T_{2}$, and changes in these parameters, with the goal of achieving continuous real‐time temperature imaging that can track rapid changes in temperature. This approach enables flexible reconstruction from variable window widths and from overlapping windows, which enable the same high‐precision scan to be used to measure both slower heating such as hyperthermia and faster heating such as ablation with focused ultrasound or laser applicators. The present work focused on the latter higher‐temperature applications. For slow heating such as in hyperthermia that may benefit significantly from qRF‐MRF's high precision, further development and corrections may be needed to mitigate confounds such as respiration and $B_{0}$ field shifts that can obscure small and diffuse heating. By incorporating temporal temperature curves or basis functions in the dictionary, temperature maps could even be reconstructed from the entire dataset all at once, which may be advantageous for measuring overall thermal dose.

While spiral readouts have been used previously for MR thermometry,^7^, ^8^, ^9^ they can require trajectory calibrations such as the trajectory measurement used here. One indication of the need for further calibration was the ring‐like temperature errors observed in the phantom precision experiment (Figure 5), which were not present in simulation. We ascribe these to residual or dynamic trajectory errors or $B_{0}$ eddy current terms, and they are likely also present in the in vivo precision maps but harder to appreciate due to higher overall errors in vivo and the irregular shape of the human head. Spirals are in general more sensitive to off‐resonance than 2DFT scans, off‐resonance effects can be mitigated with spiral in‐out sampling,^8^ or corrected using general off‐resonance correction methods^31^ or chemical shift‐corrected thermometry.^9^ Off‐resonance corrections may in particular be required for application of qRF‐MRF to imaging temperature near metal such as DBS electrodes.

Our validation results are potentially limited by the choice of 2DFT GRE thermometry as a benchmark. One limitation is that 2DFT GRE thermometry is based on NMR signals, rather than a different physical mechanism, such as leveraged by fiber optic temperature probes. 2DFT GRE thermometry was selected as the benchmark measurement in this work because it is well‐established, easily replicated, and is used clinically for PRFS thermometry. This choice further enabled voxel‐by‐voxel comparison of temperature maps rather than requiring the careful placement and localization of a probe, and it enabled comparison of temperatures both while focused ultrasound was switched on and afterward, which to our knowledge is not possible with fiber optic probes.^32^ A second limitation is that, while 2DFT is a reasonable benchmark, it is not a state‐of‐the‐art MR thermometry sequence, and it is possible that more recently described sequences could have provided more precise and accurate benchmark measurements, or measurements with finer spatiotemporal resolution.

We further demonstrated that qRF‐MRF thermometry enables reconstruction of linewidth maps. In vivo, these maps reflected areas of high static off‐resonance above the sinuses and ear canals, as well as tissues with high iron concentration. Linewidths will also be wider in heated regions and may, for example, enable super‐resolution reconstruction via their relation to local temperature gradients. Knowledge of linewidth may also be useful in unwrapping temperature maps. qRF‐MRF has been previously demonstrated for quantitative susceptibility mapping^33^ while GRE PRF‐shift thermometry has been leveraged to generate susceptibility‐weighted images that can elucidate subthalamic structures and are inherently registered to temperature maps.^34^ The qRF‐MRF sequence proposed here could provide the same capability but with higher SNR and precision compared to 2DFT GRE. At the same time, we note that in vivo precision was worst in regions with wide linewidths, which also had slightly lower inner products, suggesting that further improvements in precision may be possible by improved linewidth modeling.

In principle, sensitivity to $T_{1}$ and/or $T_{2}$ could be re‐introduced in the qRF‐MRF sequence by incorporating periodic inversion pulses or variable flip angles, and it should then be possible to reconstruct temperature maps based on each of these parameters or combinations of them at different temporal scales. Indeed, even with a small and constant flip angle, the amplitude signal in the heated voxel in Figure 9D appeared to be modulated by changes in $T_{1}$ and/or $T_{2}$ with heating. Future work will assess the scan's current ability to produce dynamic $T_{1}$ and/or $T_{2}$ maps, and how to best enhance that ability using periodic inversion pulses or variable flip angles. Increasing sensitivity to changes in $T_{1}$ and/or $T_{2}$ with heating could be used to, for example, retain a high 2‐3 s frame rate for PRF shift‐based thermometry in aqueous tissues, while simultaneously reconstructing $T_{1}$‐ and/or $T_{2}$‐based temperature maps in fatty tissues.^17^ Enhanced $T_{1}$‐weighting to separate the fat signal in each voxel^35^ or spectral–spatial or binomial excitation to suppress fat signals may be needed in order to avoid temperature errors caused by fat when applying qRF‐MRF thermometry in the body. The ability to reconstruct $T_{1}$ and/or $T_{2}$ maps from the qRF‐MRF data could further enable synthesis of $T_{1}$‐weighted and $T_{2}$‐weighted images that would be self‐registered to the temperature maps.

While many temperature mapping applications such as transcranial focused ultrasound ablation can be guided by single‐slice imaging,^36^ volumetric coverage is usually desirable to more completely capture the heated region and monitor for unintended off‐target heating. The current work focused on proving the concept of qRF‐MRF thermometry, and future work will focus on achieving volumetric coverage and faster frame rates with qRF‐MRF that are commensurate with other state‐of‐the‐art fast thermometry methods, and validating it against those methods. There are multiple possible approaches to increase volume coverage, including applying phase encoding in the slice dimension,^8^ applying adjacent‐slice simultaneous multislice encoding,^37^ or interleaving the collection of full‐time windows for each slice. As demonstrated in vivo, faster frame rates can be achieved by smoothly trading off temperature precision for frame rate. Further improvements may be achieved by optimizing the number of spiral interleaves and eliminating all dead time in the sequence. Further reductions in the spiral readout duration could be made by maximizing the spiral gradient slew rate; a maximum slew of 90 mT/m/ms was used here. Denoising can be applied to reduce temperature uncertainty in GRE PRF‐shift thermometry.^38^ The aliasing in reconstructed MRF images makes them less well‐suited for denoising, but denoising could be applied to qRF‐MRF frequency maps, or to frequency difference maps. Kalman filtering may also be applied to reduce aliasing and temperature errors in qRF‐MRF thermometry.^39^

Finally, further work is needed to achieve online reconstruction of qRF‐MRF temperature maps. This work used offline reconstruction in MATLAB with no optimization to minimize compute time. Using an Apple MacBook Pro with an M3 Max CPU and 128 GB RAM (Apple Inc, Cupertino, CA, USA), CG reconstruction of 20‐channel data took 90 s per 220 TRs of data jointly reconstructed, while gridding reconstruction took 9.6 s per 220 TRs. Matching to calculate frequency maps then took another 15 s. Implementing the reconstruction in real time will require a combination of more capable compute resources, including GPUs, and parallelization and data and dictionary compression. For example, just parallelizing the CG NUFFT's across the twenty receiver channels should reduce the CG reconstruction time by almost 20×.

## 6 CONCLUSION

A quadratic phase RF‐MR fingerprinting (qRF‐MRF) method for temperature imaging using the proton resonance frequency shift was reported and tested in heated phantom and unheated in vivo experiments. Results demonstrated the feasibility of using qRF‐MRF for real‐time, in vivo MR thermometry with considerably higher precision than a frame rate‐matched 2DFT GRE scan. The qRF‐MRF reconstruction can be used with existing motion correction and artifact reduction methods for PRF‐shift thermometry.

## FUNDING INFORMATION

This work was supported by NIH grants R01 NS120518 and R01 EB028773 and Siemens Healthineers.

## CONFLICTS OF INTEREST

CWRU MRI lab receives research support from Siemens Healthineers.

## DISCLAIMER

Certain commercial equipment, instruments, software, or materials are identified in this paper in order to specify the experimental procedure adequately. Such identification is not intended to imply recommendation or endorsement by NIST, nor is it intended to imply that the materials or equipment identified are necessarily the best available for the purpose.

## Supporting information


**Data S1.** Supporting Information.

## Data Availability

The pulse sequence generation code is hosted at https://github.com/sjgarrow/qRF‐MRFThermometrySequences. The experimental data, images, and code from this study are available upon request to the corresponding author.
