# Supplementary material for: MR thermometry with high precision and temporal resolution by quadratic phase MR fingerprinting
Source: Magn Reson Med. 2025 Apr 28;94(3):1119–35. doi: 10.1002/mrm.30546 (PMC12202739; doi:10.1002/mrm.30546)
Supplement: Supplementary file 1 — Data S1. Supporting Information. [file MRM-94-1119-s001.pdf]

Supporting Information: MR Thermometry with High Precision and Temporal Resolution by Quadratic Phase MR Fingerprinting

Sarah J Garrow[1], Kristen Zarcone[1], Kathryn E Keenan[2], Rasim Boyacioğlu[3], Mark Griswold[1,3], and William A Grissom[1,3]

[1]Biomedical Engineering, Case Western Reserve University, Cleveland, OH, USA

[2]National Institute of Standards and Technology, Boulder, CO, USA

[3]Department of Radiology, Case Western Reserve University, Cleveland, OH, USA

April 4, 2025

Address correspondence to:

William A Grissom, Ph.D.

BRB 333

2109 Adelbert Road

Cleveland, OH 44106

wag57@case.edu

This work was supported by NIH grants R01 NS120518 and R01 EB028773 and Siemens Healthineers.

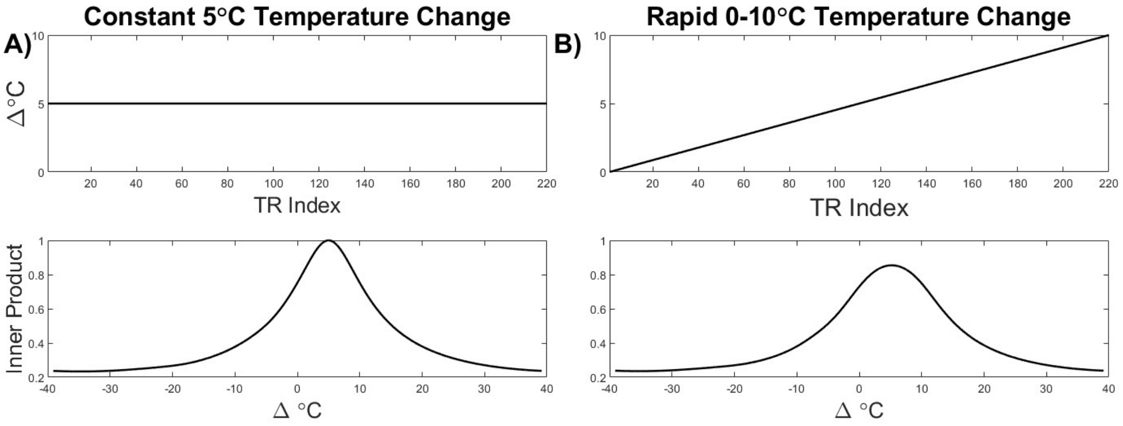

Supporting Information Figure S1: Simulated within-window heating and corresponding inner products across temperatures. A) Shows a constant 5° C temperature change over a 220 TR window, which matched to a temperature of 5° C. B) Shows a linear heat ramp from zero to 10° C corresponding to rapid heating within the 2.2-second window. Because qRF-MRF signal is weighted equally by each TR in the window, the inner product spectrum broadens but the peak remains at 5° C.

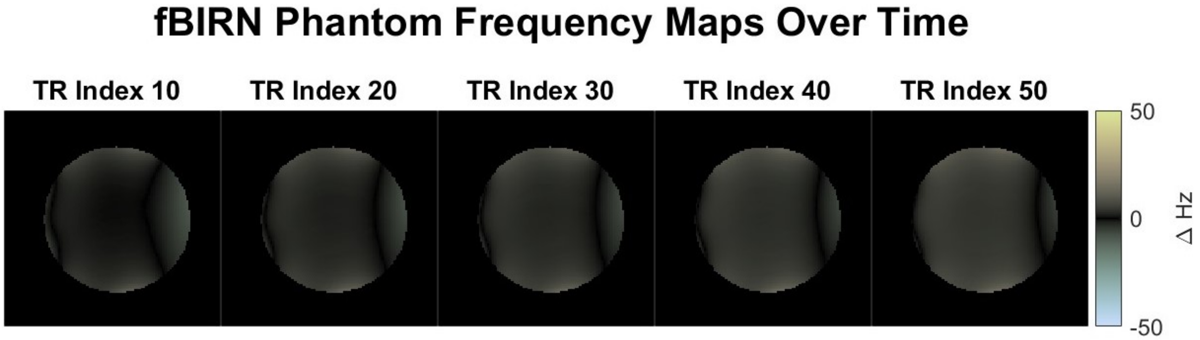

Supporting Information Figure S2: fBIRN phantom frequency maps at 5 timepoints through the temperature precision experiment. The frequency varies from -7 to +10 Hz, with a mean of 2.3 Hz and a standard deviation of 2.4 Hz; the dark band on the right corresponds to 0 Hz.

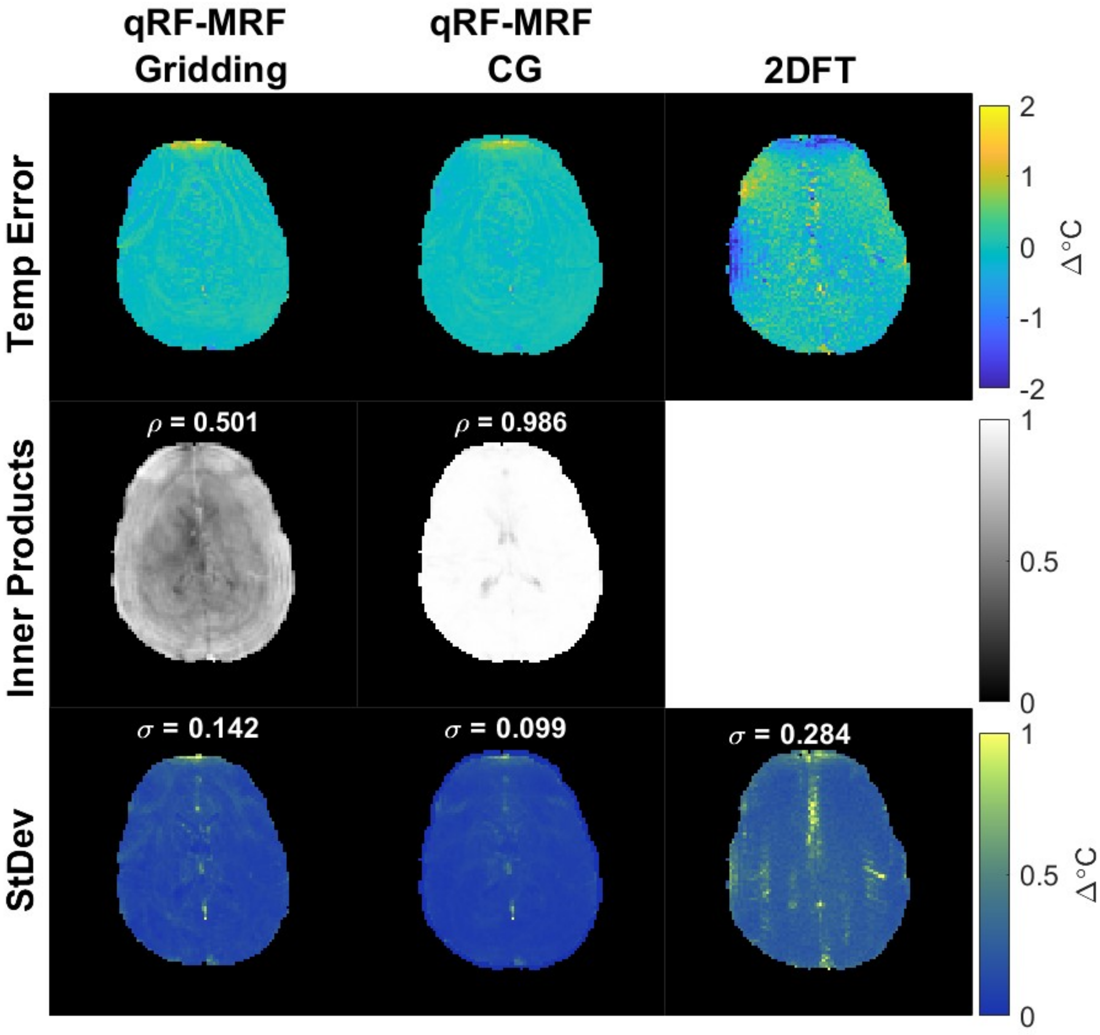

Supporting Information Figure S3: Comparison of gridding versus conjugate gradient qRF-MRF reconstruction in Volunteer 2's superior slice. The temperature error in a single TR index (top row) is decreased using iterative reconstruction vs gridded reconstruction. The standard deviation within the head in this single TR index is reported above each case. For qRF-MRF, the correlation (middle row) are also twice as high when using CG compared with gridding. The mean standard deviation over time (two minutes) for each case (bottom row) is reported above each method, in units of degrees Celsius.

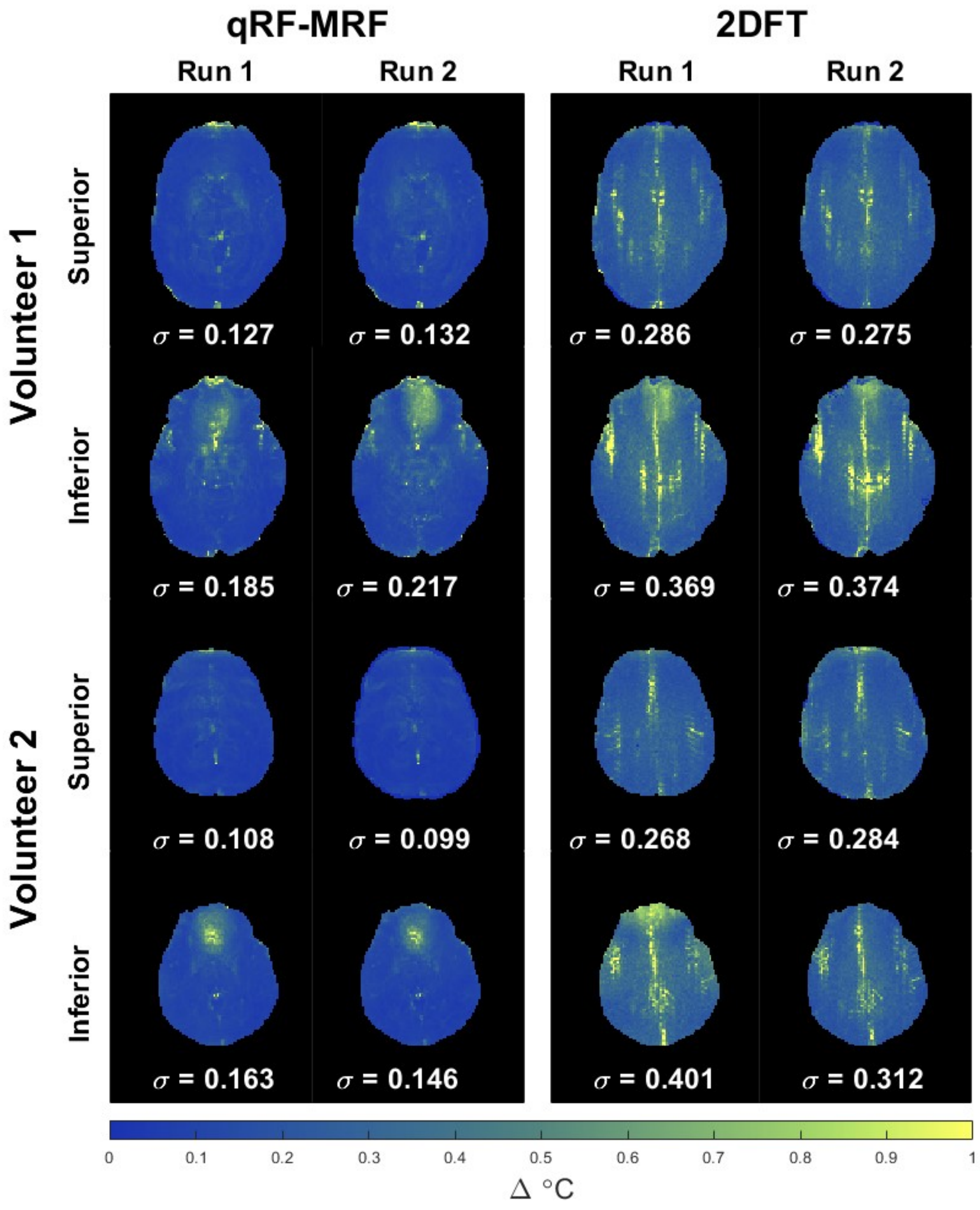

Supporting Information Figure S4: Repeated through-time temperature standard deviation scans from each volunteer and slice. The overall standard deviations are reported for each volunteer, slice, and repetition in units of degrees Celsius. qRF-MRF maps have highest errors near the front of the brain above the sinuses. The 2DFT maps also have higher error above the sinuses but their overall highest errors are along the midline and in locations where pulsatile flow in blood vessels caused signal to alias along the phase-encoded (anterior/posterior) direction.

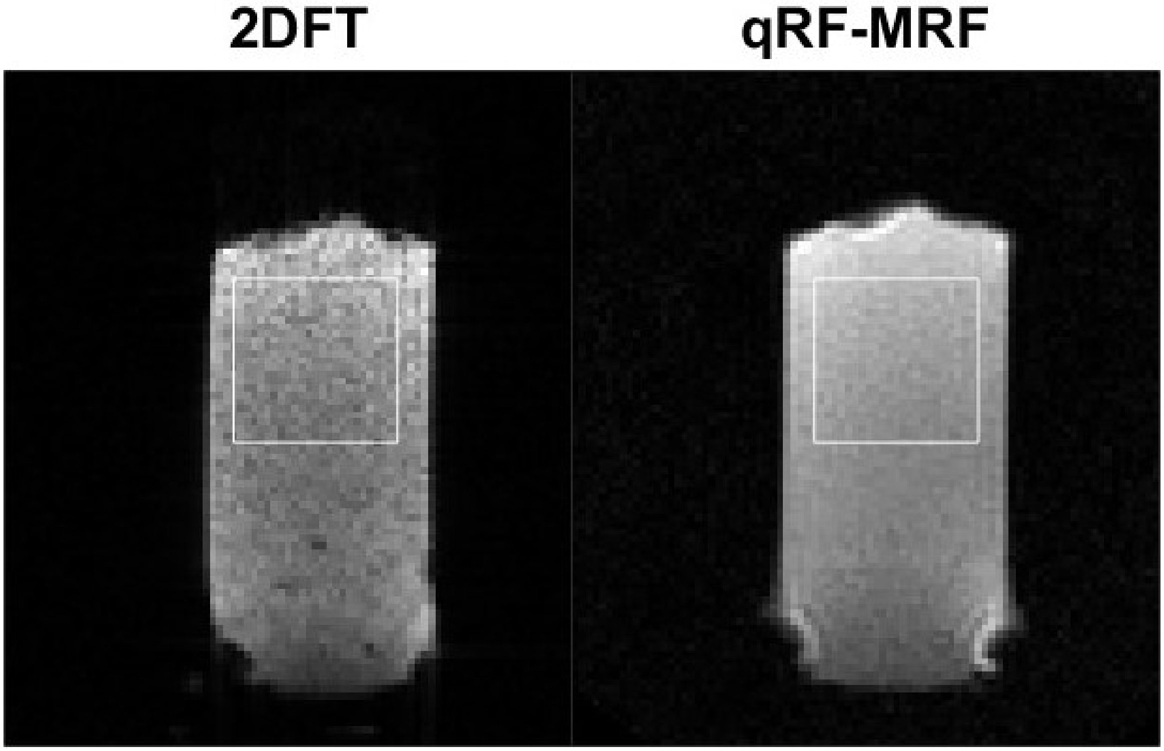

Supporting Information Figure S5: 2DFT and time-averaged qRF-MRF magnitude images for a single time window in the focused ultrasound heating experiment. The SNR of the ROI shown in the white rectangles was 11.4 for the 2DFT reconstruction and 44.3 for the qRF-MRF reconstruction. The transducer is located below the phantom, pointing upward.

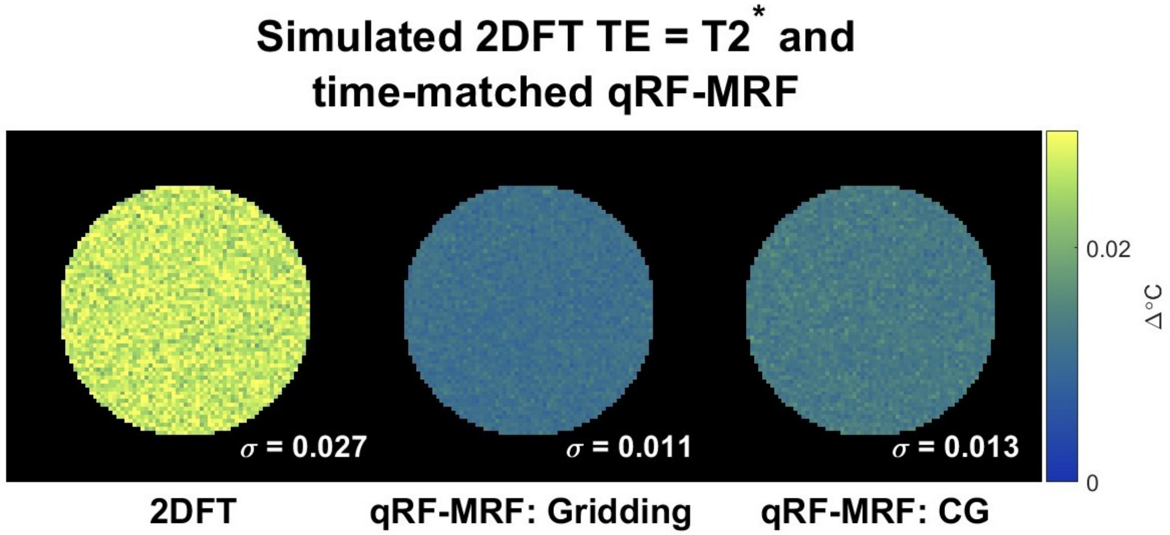

Supporting Information Figure S6: Monte Carlo-simulated temperature standard deviation maps for the 2DFT scan using TE = T_2^* = 46 ms, TR = 60 ms, and an Ernst angle of 21.6°, and qRF-MRF scans, where qRF-MRF data were reconstructed using gridding and CG. The qRF-MRF window width was 7.68 seconds (768 TRs) to match the 2DFT scan time. The overall standard deviations are reported in white, in units of °C.
